# MAIT cells and the microbiome

**DOI:** 10.3389/fimmu.2023.1127588

**Published:** 2023-02-23

**Authors:** Maisha F. Jabeen, Timothy S. C. Hinks

**Affiliations:** ^1^ Respiratory Medicine Unit, Experimental Medicine Division, Nuffield Department of Medicine, University of Oxford, John Radcliffe Hospital, Oxford, United Kingdom; ^2^ National Institute for Health Research Oxford Biomedical Research Centre, John Radcliffe Hospital, Oxford, United Kingdom

**Keywords:** MAIT cell, microbiome and dysbiosis, tissue homeostasis, airways diseases, inflammatory bowel conditions, metabolic syndromes, stem cell transplant (SCT)

## Abstract

Mucosal associated invariant T (MAIT) cells are innate-like T lymphocytes, strikingly enriched at mucosal surfaces and characterized by a semi-invariant αβ T cell receptor (TCR) recognizing microbial derived intermediates of riboflavin synthesis presented by the MHC-Ib molecule MR1. At barrier sites MAIT cells occupy a prime position for interaction with commensal microorganisms, comprising the microbiota. The microbiota is a rich source of riboflavin derived antigens required in early life to promote intra-thymic MAIT cell development and sustain a life-long population of tissue resident cells. A symbiotic relationship is thought to be maintained in health whereby microbes promote maturation and homeostasis, and in turn MAIT cells can engage a TCR-dependent “tissue repair” program in the presence of commensal organisms conducive to sustaining barrier function and integrity of the microbial community. MAIT cell activation can be induced in a MR1-TCR dependent manner or through MR1-TCR independent mechanisms *via* pro-inflammatory cytokines interleukin (IL)-12/-15/-18 and type I interferon. MAIT cells provide immunity against bacterial, fungal and viral pathogens. However, MAIT cells may have deleterious effects through insufficient or exacerbated effector activity and have been implicated in autoimmune, inflammatory and allergic conditions in which microbial dysbiosis is a shared feature. In this review we summarize the current knowledge on the role of the microbiota in the development and maintenance of circulating and tissue resident MAIT cells. We also explore how microbial dysbiosis, alongside changes in intestinal permeability and imbalance between pro- and anti-inflammatory components of the immune response are together involved in the potential pathogenicity of MAIT cells. Whilst there have been significant improvements in our understanding of how the microbiota shapes MAIT cell function, human data are relatively lacking, and it remains unknown if MAIT cells can conversely influence the composition of the microbiota. We speculate whether, in a human population, differences in microbiomes might account for the heterogeneity observed in MAIT cell frequency across mucosal sites or between individuals, and response to therapies targeting T cells. Moreover, we speculate whether manipulation of the microbiota, or harnessing MAIT cell ligands within the gut or disease-specific sites could offer novel therapeutic strategies.

## Introduction

The seminal discovery that the mucosal associated invariant T (MAIT) T cell receptor (TCR) recognizes riboflavin metabolites derived from bacteria, mycobacteria and fungi (1), revealed a prime role in sensing and responding to the microbiome at mucosal surfaces. The MAIT TCR is a semi-invariant TCR-α chain (typically TRAV1-2-TRAJ33 or TRAV1-2-TRAJ12 or TRAV1-2-TRAJ20), predominantly associated with the β-chains TRBV20 or TRBV6 in humans and TRBV19 or TRBV13 in mice and specifically recognizes the naturally-occurring activating ligand 5-(2-oxopropylideneamino)-6-dribityllumazine (5-OP-RU) presented on MHC-related protein 1 (MR1) (2). Whilst initially these cells were understood to play a role in antimicrobial host defense, the more recent discoveries of separate antiviral and ‘tissue repair’ responses have revealed a more nuanced complexity in their functional repertoire. Nonetheless the microbiome remains absolutely essential to the development and peripheral expansion of MAIT cells as a source of TCR ligand, such that the nature of the early life microbiome can mediate life-long changes in the MAIT cell repertoire. In this review we therefore make a specific focus on the role of the microbiome in the ontogeny of MAIT cells. We then review how microbial dysbiosis, often marked by compositional shifts in specific phyla, alongside changes in intestinal permeability and inflammatory cytokine milieus, are together involved in the potential pathogenicity of MAIT cells. Though human data on the influence of MAIT cell deficiency on the microbiome are relatively scarce, we explore the insights given by specific clinical instances of acquired MAIT cell deficiency, in particular those of hematopoietic stem cell transplantation and of human immunodeficiency virus (HIV)-induced MAIT cell loss, which provide experimental windows into MAIT cell biology. We review human data from the gut, lung and skin, which comprise the body’s largest barrier surfaces, and conclude with thoughts on the potential for therapeutic manipulation of the microbiome or MAIT cells populations directly.

## Ontogeny of MAIT cells

Whilst conventional CD4+ or CD8+ T cells exit the thymus as naïve cells, gaining effector functionality following antigen exposure in secondary lymphoid organs, MAIT cells acquire effector functions intra-thymically (3–5). We review the ontogeny of MAIT cells in mouse and human and consider how commensal derived bacterial metabolites are needed at each stage of their development and acquisition of antimicrobial functionality.

### Mouse

MAIT cells arise in the thymic cortex following the development of CD4^+^CD8^+^ double positive (DP) thymocytes possessing a T cell receptor (TCR) specific to the MR1:5-OP-RU complex, surveying their vicinity for subsequent positive selection of MR1-expressing cells (6). Using a murine bone marrow chimera model and single cell RNA sequencing, it has been shown that positive selection of 5-OP-RU : MR1 specific thymocytes can occur on different cell types destined for divergent outcomes. These are heat stable antigen high (HSA^hi^) precursors undergoing positive selection by thymic epithelial cells (TEC) and differentiating into naïve CD4^+^ T cells or, thymocytes selected by hematopoietic cells differentiating into CD44^hi^ effector cells (7).

**Figure 1:**
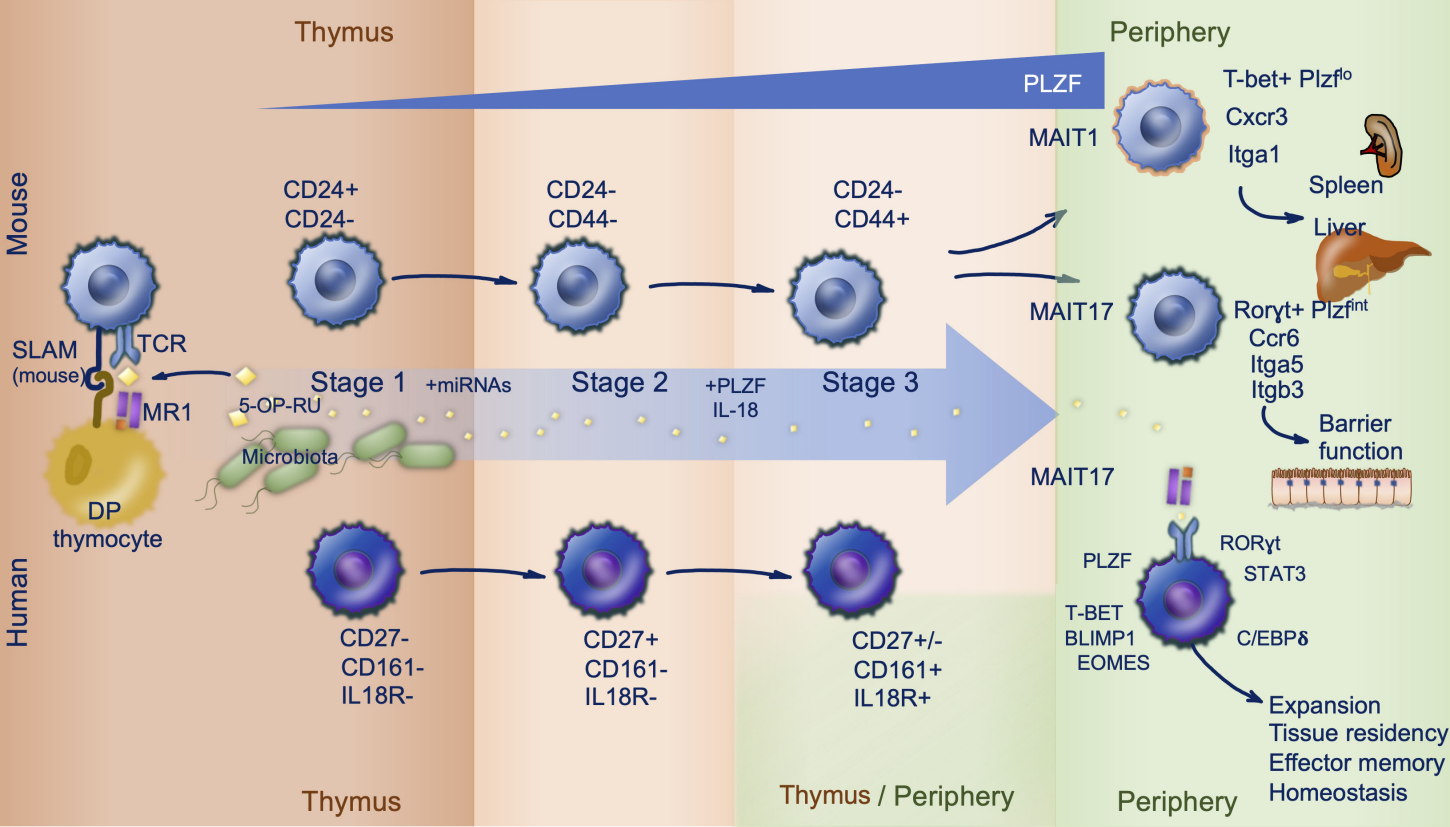
MAIT cell ontogeny. Mouse (top) and human (bottom) mucosal associated invariant T (MAIT) cell development follows three stages, largely within the thymus. Microbially derived 5-OP-RU is trafficked to the thymus and loaded onto MR1 expressed on double positive (DP) thymocytes. MAIT cells are subsequently positively selected on DP thymocytes in a process reliant upon signaling lymphocytic activation molecule (SLAM) interactions in mice (stage 1). During stage 2 and 3 MAIT cells acquire effector functions and their prototypic phenotype driven by PLZF expression. This occurs intrathymically in mice, but in human stage 3 can also occur peripherally. Distinguishing markers and important co-factors regulating differentiation are shown.

Regardless of their mode of selection there is resultant expression of the survival factor Bcl2 with induction of Ccr7 and loss of Ccr9 expression to suggest migration of these cells from the thymic cortex to the medulla. Following positive selection in mouse and human, MAIT cells progress through three stages of intrathymic development summarized in **Figure 1**. Like iNKT cells (8), effector differentiation of murine MAIT cell precursors selected by hematopoietic cells in the thymus is reliant upon expression of the signaling lymphocyte activation molecule (SLAM) adaptor protein (SAP) (4, 7, 9). This shared characteristic reflects ZBTB16 expression (encoding the master transcription factor PLZF) in both cell types, conferring innate-like functionality. PLZF suppresses the naïve T cell program and is required for expression of effector genes, alongside CD44 expression (3, 4). There is concurrent downregulation of Bach2 (a transcription factor seen in conventional T lymphocytes) and then Klf2, involved in regulating thymic egress (4). The population of naïve MR1-restricted T cells, having undergone positive selection on MR1 expressing TECs, patrol secondary lymphoid organs whereas their counterparts selected on MR1 expressing hematopoietic cells undergo proliferation and differentiation in preparation for migration to peripheral tissue to support mucosal immunity (7).

In mice two clear MAIT cell subsets with distinct effector properties are identified by their expression factors T-bet (MAIT1) and RORγt (MAIT17) enabling them to readily produce IFN-γ and IL-17 respectively (7). Whist single cell RNAseq has been informative in describing early transcriptional event in MAIT cell development, the signaling pathways and mechanisms responsible for adoption of a MAIT1 versus MAIT17 phenotype are yet to be fully elucidated. It has been speculated that choice of lineage may be directed at random by positively selected MAIT cell precursors, shaped by environmental cues such as cytokines or relate to TCR affinity for the selecting ligand but this remains to be confirmed experimentally (10). MAIT1 and MAIT17 cells express distinct patterns of chemokine receptors and integrins; MAIT1 cells express Cxcr3 and Itga1, while MAIT17 cells express Ccr6, Itga5 and Itgb3 and respond more efficiently to TCR-stimulation (7, 11, 12). PLZF expression in MAIT cells confers a broad tissue homing capacity whereas the transcription factors T-bet and RORγt likely fine tune tissue targeting, promoting residency of MAIT1 cells preferentially in spleen and liver, and MAIT17 cells in barrier tissue including the lung, skin and gut (11, 12).

### Human

Mature T cell development begins *in utero* in humans. MAIT cell precursors appear early during gestation at low frequencies in the thymus and cord blood (5, 13). MR1 is expressed on hematopoietic double positive thymocytes suggesting a similar manner of selection to mouse MAIT cells (14). In human cord blood a minority of Vα7.2^+^CD161^+^ have high avidity for the MR1:5-OP-RU complex; only these cells go on to acquire a memory phenotype in the first few weeks of life and expand to provide the adult MAIT cell pool over the following 5 to 6 years (13). Thus, the adult MAIT cell clonal size is antigen driven and results in a restricted TCR repertoire in adults (13). Key similarities and differences between mouse and human MAIT cell development are discussed below.

As seen in mouse, human MAIT cells preferentially locate to non-lymphoid tissue. Thymic MAIT cells express tissue-homing molecules including CCR6, CCR5 and CXCR6 (4). In addition, as they mature, thymic MAIT cells upregulate the transcription factors C/EBPδ (4), important for MAIT cell trafficking (15), and RUNX3 (4), also required by conventional CD8^+^ tissue resident memory cells to establish niches in diverse tissue environments (16). The shared transcription factors PLZF (ZBTB16), RORγt (RORC) and T-bet (TBX21) are induced differentially in both mouse and human during thymic development (5), but expression of SAP is not necessary for human MAIT cells (5, 7). Mature tetramer positive (MR1:5-OP-RU restricted) thymocytes express PLZF, CD161 and IL-18Rα. However, unlike thymic MAIT cells in mouse, which display a memory phenotype at the point of egress, mature thymic and cord blood MAIT cells in human are negative for the memory marker CD45RO (5, 13). In addition, human MAIT cells are largely T-bet^+^RORγt^+^ as they exit the thymus (12) whereas these transcription factors are expressed mutually exclusively in mouse (giving way to MAIT1 and MAIT17 cells) (5). Peripheral MAIT cell expression of transcriptions factors Eomesederin (EOMES) and Blimp-1 (PRDM1) further support type-1 responses, whereas STAT3 supports type-17 responses. Human thymic MAIT cells possess cytotoxic capacity (4) but limited ability to secrete TNF and IFN-γ in response to PMA and ionomycin stimulation when compared to peripheral blood MAIT cells (3). Therefore, in human MAIT cells terminal maturation appears to occur in the periphery following birth suggesting additional peripheral mechanisms support acquisition of full effector functionality.

## Microbes and MAIT cells at barrier sites: Development and homeostasis

The microbiome has a fundamental role in the induction, development, and homeostatic function of the host immune system. A symbiotic relationship between the host immune system and microbiota is required to balance regulatory pathways conferring tolerance to innocuous antigens and protective immunity against pathogens (17).

Following their development in the thymus MAIT cells are equipped with a transcriptional program and homing markers to support tissue residency. They localize to sites including the oropharynx, respiratory and GI tracts, skin and female genital mucosa, also hosting uniquely adapted microbial communities (18). MAIT cells thereby occupy a prime position for crosstalk with commensal microorganisms which uniquely synthesize riboflavin at mammalian barrier surfaces (18). The co-evolution of MR1 and TRAV1, and accumulation of MR1 mutations in species following loss of TRAV1, supports the idea that the main function of MR1 is to present antigen to MAIT cells (19). By extension this provides an insight into conserved mechanisms through which barrier surfaces can imprint mucosal immunity. These interactions are further shaped by cellular networks, environmental and metabolic factors within the microenvironment (20). With broad anti-bacterial specificity and a capacity for tissue repair, MAIT cells may be key in restoring homeostasis following infection or tissue injury thereby offering protection from invading pathogens and preserving the microbiome.

### Microbes and MAIT cell development

MAIT cell reliance on the microbiome is evidenced by their relative deficiency in the periphery (21) and thymus (3) of germ free (GF) mice, compared with specific pathogen free animals. This pattern of deficiency is similar to other innate and memory T cell populations in GF and antibiotic treated mouse models (22). Metabolites from riboflavin-synthesizing commensals are needed for most stages of MAIT intra-thymic development and subsequent peripheral expansion (3, 23). Riboflavin metabolites secreted by the microbiota travel rapidly to the thymus. In fact 5-OP-RU is trafficked and detected in the thymus within an hour of topical or oral administration (23). MAIT cell development can be promoted following mono-colonization of GF mice with riboflavin synthesizing bacterial species such as *Proteus mirabilis* or *Escherichia coli*, but not with species deficient in this biosynthetic pathway such as *Lactobacillus johnsonii* (21, 23). Using mutant *E. coli* strains deficient in riboflavin enzymes either upstream (ΔRibD) or downstream (ΔRibE) of 5-A-RU to colonize GF mice has identified the essential role for RibD, and thus 5-OP-RU, in thymic MAIT cell development. The non-stimulatory MR1-ligand Acetyl-6-formyl-pterin (Ac-6-FP) cannot support MAIT cell development (23). Following recolonization of GF mice, there is selective restoration of RORγt+ MAIT17 cells reliant on TCR-triggering for proliferation and function (23). Of note, GF mice retain a small residual population of thymic MAIT cells whereas Mr1^-/-^ are completely lacking in MAIT cells strongly supporting an essential role for MR1 in positive selection of these cells (7).

Microbial recolonization of adult GF mice restores thymic MAIT cell development, but fails to populate peripheral tissue such as the skin (21) or lung (23) with newly differentiated MAIT cells. There is a narrow neonatal window (within first 3 weeks of life) when recolonization of GF mice can restore the MAIT cell population (21). In addition, topical administration of 5-OP-RU is sufficient for MAIT cell development and skin homing in neonates but not adults (21, 23). Thus, adult MAIT cell development is reliant on microbiome-derived co-stimulation. However, preservation of MAIT cells in MyD88- and TLR3- deficient mice rules out TLR or IL-1 receptor family members (IL-1, IL-18 or IL-33) as likely drivers (23). A further explanation for this temporally-restricted reconstitution of MAIT cell populations may be competition for a shared niche imposed by similar innate T cell subsets. MAIT cell frequencies positively correlate with mouse and human iNKT cells (13, 24) and γδT cells (24), all sharing overlapping functions and a reliance on the microbiome. Competitive regulation of individual populations is supported by increased frequency of iNKT and MAIT cells seen in Tcrd-deficient mice (21) and increased splenic and thymic MAIT cells in Cd1d-deficient mice (3). In humans, the R9H mutation in MR1prevents its binding to 5-OP-RU but retains affinity for Ac-6-FP (25); in a rare patient with a homozygous R9H mutation in MR1, MAIT cell deficiency is observed with an expanded γδT cell (Vδ2+) population, again suggesting a compensatory interaction between innate T cell subsets (25). MAIT cells, iNKT and γδT cells do not compete for the same antigen, thus competition for immunological space may be imposed through alternative mechanisms orchestrated by immunoregulatory cytokines (e.g. IL-7, IL-15) (26–28), or host and dietary metabolites regulating shared transcriptional pathways (e.g. *via* the aryl hydrocarbon receptor) (20, 29). It is yet to be determined if population pressures and temporal restrictions apply to restoring human MAIT cell frequencies. Partial reconstitution of MAIT cells is seen following allogenic hematopoietic stem cell transplantation (HSCT) and correlates with the diversity of gut microbiota (30). It is tempting to speculate that the microbiome offers a key to regulating MAIT cells, however it has not yet been fully elucidated how this interaction could be skewed by neighboring innate T cells or the effects of age and disease.

The microbiome is diverse and heterogenous, varying between mucosal sites with distinct microenvironments. A large *in vitro* screen of microbiota-associated bacterial species found that the capacity to stimulate MAIT cells correlated with riboflavin secretion as measured by mass spectrometry (31). High stimulator species belonged to Bacteroidetes and Proteobacteria phyla, whereas Actinobacteria and Firmicutes were poor stimulators. Vβ2^+^ MAIT cells were most activated. Conventional human T cell subsets were able to present MR1-ligand but induced a weaker cytokine response compared to professional APCs. This suggests a capacity for MAIT cells to discriminate between members of the microbiota by TCR signal strength based on antigen load and presenting cell (31). Microbial diversity has been shown to reduce MAIT cell activation *in vitro*, correlating with net riboflavin secretion in a human intestinal model community. Higher diversity resulted in greater riboflavin consumption and thus less antigen presentation to MAIT cells. Interestingly, introducing microbial stress through environmental acidification reduced activation by impairing availability of riboflavin (32). MAIT cells have also been shown to exhibit microbe-specific responses to bacterial and fungal organisms with differential TCR β-chain bias and MR1-dependent activation, suggesting a further dimension of functional heterogeneity (33).

Microbial diversity varies by tissue, and notably between health and disease, typically marked by dysbiosis with reduced diversity. As discussed below, this may be a mechanism through which MAIT cells contribute to pathology and equally offer a therapeutic opportunity to manipulate MAIT cell function. It is worthwhile considering barrier homeostasis alongside microbial diversity. Pathogen invasion disrupts the mucosa and induces an inflammatory response. Co-stimulation of MAIT cells *via* TCR and cytokine has been shown *in vitro* to engage the full antimicrobial repertoire in MAIT cells (34). Therefore, where MAIT cells are implicated in disease pathogenesis it is important to consider any changes to the microbiome in parallel. Further studies, particularly in human, are needed to address this and consider specific mechanisms which shift MAIT cell function from homeostatic to pro-inflammatory.

### Microbes and tissue repair

MAIT cells can engage a “tissue repair” program associated with accelerated wound repair in the context of commensal organisms. We and others have described the transcriptome of activated MAIT cells following transcriptomic analysis of MR1:5-OP-RU tetramer positive cells in mouse and human (34–36). Alongside expected pro-inflammatory responses, we identified a TCR-mediated and activation-driven expression of the tissue repair program previously reported in murine skin homing H2-M3 restricted Tc17 cells induced by commensal flora and accelerating repair in an epithelial wound model (35, 37). Key genes expressed in both species included TNF, CSF2, HIF1A, FURIN, VEGFB, PTGES2, PDGFB, TGFB1, MMP25, and HMGB1 (35). Accelerated wound healing could be observed in an intestinal epithelial cell line system following treatment with supernatants from TCR-stimulated MAIT cells and blocked with anti-MR1 antibodies (36). This MAIT tissue repair program is observed following TCR ligation but not cytokine mediated stimulation alone (34, 36). Thus, similarly to H2-M3 restricted Tc17 cells in mouse skin (37) and γδT cells in the lung and gut (38–40), MAIT TCR signaling appears to play a role in tissue homeostasis.

Murine skin-resident MAIT cells also engage a distinct tissue repair transcriptional signature (21) reminiscent of H2-M3 restricted Tc17 cells reactive to *S. epidermidis* derived N-formylated peptides (37). To unpick the role of MAIT cells from H2-M3 restricted Tc17 or γδT cells, given their overlapping properties, Tcrd^-/-^ mice and *S. epidermidis* strain incapable of inducing H2-M3 Tc17 cells were utilized (21). This revealed a MAIT cell-dependent tissue repair response to *S. epidermidis* with accelerated epidermal tongue length growth in a skin punch biopsy model compared with MAIT cell deficient Mr1^-/-^Tcrd^-/-^ mice. A further observation from this study was that direct topical application of the MAIT cell ligand 5-OP-RU prior to skin injury, in the presence or absence of additional cytokines, was sufficient to induce local expansion of MAIT cells and accelerate tissue repair (21). The importance of this role at human barrier sites and mechanisms through which MAIT cell mediators might exert their homeostatic function on the local environment is yet to be elucidated.

Murine studies have demonstrated reduced intestinal microbial diversity in MR1 deficient animals, which may result from altered IL-17A signaling downregulating tight junction protein expression (41). In non-obese diabetic (NOD) mice deficient in MR1 there is impaired intestinal barrier integrity (42). Further work is needed to understand if human MAIT cells can shape the composition of the healthy microbiome. As discussed below, in disease states this is likely to be closely intertwined with barrier integrity and its effects on the microbiota.

### Tissue localization and MAIT cell phenotype

Microbiome-derived signals are likely to contribute to the establishment of tissue resident MAIT cell populations as murine lung and skin MAIT cell frequencies at steady state are cage dependent (21). It is unknown if this is shaped by antigen load or other innate signals. Cytokines appear to have a varied role; in skin IL-23 signaling is necessary for sustaining a MAIT17 cell population (21), yet in the lung normal MAIT cell frequencies are maintained not only in Il23^-/-^ mice but also Ifng^-/-^, Il6^-/-^, Il18^-/-^ and Il12^-/-^ deficient animals (43). Cytokine reliance may be imprinted in the thymus as Il23r expression is higher in the MAIT17 thymic subset (4, 44). Tissue MAIT cells undergo terminal differentiation in tissue with unique transcriptomic programs observed between the lung versus the spleen and liver (11), or indeed skin compared with spleen, lung and liver (21).

In humans, peripheral blood MAIT cells respond differently from tissue-derived MAIT cells originating from intestinal mucosa (45), oropharynx (46), nasopharynx (47), lung (48) and female genital tract (49) following TCR ligation, also suggesting tissue-specific imprinting. Colonic MAIT cells acquire a primed phenotype, compared with their peripheral blood counterparts, proportionately to accumulation of antigenic metabolites derived from the microbiome (50). Overall, these adaptive mechanisms are speculated to be favorable for long term residency in tissue. In parabiotic pairs most spleen, liver and lung (except some RORγt^-^ cells) MAIT cells did not recirculate over 5 weeks, implying persistent tissue residency (11). In human, whether MAIT cells are permanently resident or leave tissue and recirculate remains unclear. MAIT cells from matched thoracic duct lymph and blood samples have a shared TCR repertoire but are CCR7^-^, this could indicate transit through tissue between the two compartments or a CCR7-independent migration mechanism (51). The tissue residency markers CD69 and CD103 are widely expressed by MAIT cells at mucosal surfaces, but rare in blood MAIT cells (20). Therefore, there may be a small pool of recirculating cells in health, although its role in disease is not known.

## Microbial dysbiosis and MAIT cells in immune mediated diseases

**Figure 2:**
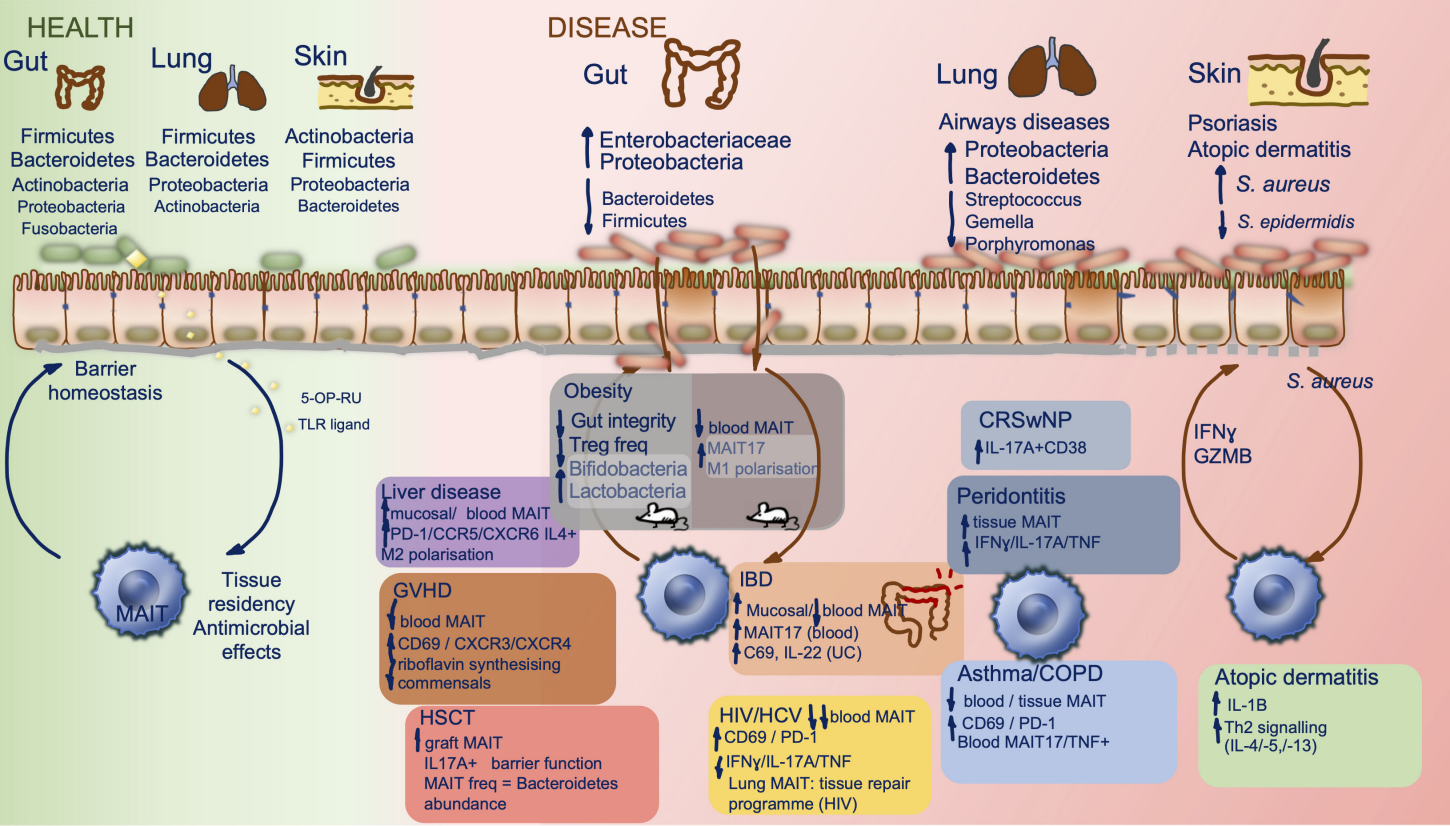
MAIT cells and the microbiome in health and disease. Constituents of the human microbiome in health, at different barrier sites, and interactions with tissue resident MAIT cells are shown (left). Key compositional shifts in human microbiota and their resultant effects in a range of disease states are summarized by barrier site (right), data are derived from human studies except within obesity where mouse studies have been included and highlighted within the figure. There is relative paucity of data on direct MAIT cell effect on the microbiome. CRSwNP, chronic rhinosinusitis with nasal polyposis; COPD, chronic obstructive pulmonary disease; GVHD, graft versus host disease; HSCT, hematopoietic stem cell transplant; HCV, hepatitis C virus; HIV, human immunodeficiency virus; IBD, inflammatory bowel disease.

MAIT cell dysfunction and dysbiosis often feature together in immune mediated diseases driven by autoimmune, atopic, and metabolic processes, alongside chronic infection. We review how MAIT cell-microbial interactions change from homeostatic to potentially harmful within this context and summarise this in **Figure 2**. Conditions characterised by shifts in the gut microbiome that provide insight into the potential symbiosis between microbiota and MAIT cell biology are considered first, before exploring other mucosal niches and their potential effects on MAIT cell phenotype. MAIT cell-microbiome interactions in specific autoimmune conditions is reviewed more extensively elsewhere (52),

### The gut microbiome and MAIT cells

The gut microbiome has been most widely studied in health and disease. It is estimated that the bacterial density of the colon is 10^11^-10^12^/milliliter making it the most densely populated microbial habitat in the human body (53). Human gut microbiota is composed of Firmicutes, Bacteroidetes, Actinobacteria, Proteobacteria, Fusobacteria, and Verrucomicrobia, with Firmicutes and Bacteroidetes accounting for 90% of total species (54). Its principal function is to protect against colonization of exogenous pathogens and potentially pathogenic indigenous organisms, through competition for finite nutrients and modulation of the host immune response (55). Throughout life the gut microbiota is required for the development of innate and adaptive arms of the immune response, providing education in host/pathogen discrimination and sustaining barrier homeostasis (55). As discussed, MAIT cell development is reliant on the gut microbiome. Bacteroidetes, highly abundant in the gut, are the strongest stimulators of MAIT cells and likely further influence their phenotype in the intestinal mucosa (31). In dysbiosis (related to localized pathology or systemic inflammation) MAIT cells, enriched in the lamina propria, are likely to be one of the first cells exposed to translocated gut microbes and inflammatory signals following the resultant barrier compromise, however their direct role in pathological processes caused by dysbiosis is yet to be fully elucidated.

Most mechanistic studies exploring MAIT cell-microbiome interactions have utilized animal models, often comparing to MR1^-/-^ strains devoid of MAIT cells. The fecal microbiota in MR1^-/-^ mice is distinct from wild-type animals with unique organisms belonging to Bacteroidaceae, Desulfovibrionaceae and unclassified Burkholderiales families, conferring greater overall richness. It is also resistant to antibacterial killing and *Clostridium difficile* colonization (56). Furthermore, intestinal barrier function has been reported to be compromised in the absence of MR1 (41, 42). Gut microbiota vary between mouse strains (57) and to date no studies have compared animals with MAIT cell deficiency or excess across common background laboratory strains to determine if shifts in microbiome are linked to their genetic background. Thus challenges arise when elucidating MAIT cell mechanisms in animal models and it is important to corroborate findings in human studies given fundamental differences not only in microbiome but also MAIT cell tissue distribution.

### Inflammatory bowel diseases

Inflammatory bowel diseases (IBD), consisting of the subtypes Crohn’s disease (CD) and ulcerative colitis (UC), are multifactorial chronic inflammatory conditions of the gastrointestinal tract associated with dramatic changes to the microbiota and local metabolic landscape (58). Several studies have investigated the relationship between genetic and immune susceptibility to IBD alongside the role of the gut microbiome in pathogenesis. This has been reviewed elsewhere (59–61). MAIT cells and their derived cytokines, particularly IL-17, have been considered as pathogenic drivers however the evidence for this is conflicting. MAIT cells are depleted in children with early onset inflammatory bowel disease under the age of six (62). Adult peripheral blood MAIT cell are activated (Ki67+) and decline in frequency in CD and UC (63, 64), unrelated to therapeutic intervention (anti-TNF or corticosteroids). There is a concurrent enrichment of ileal mucosa MAIT cells in CD and colonic mucosa MAIT cells in UC, correlating with disease activity (45). Blood MAIT cells in CD demonstrate a shift in cytokine production with greater IL-17 and reduced IFN-γ secretion following *ex vivo* stimulation (64), while UC MAIT cells upregulate CD69 (45) and secrete more IL-22 (64) and IL-17 (45). In oxazolone colitis, a murine model of UC which histologically resembles UC and is predominantly mediated by type-2 cytokines, it has been proposed that MAIT cell play a directly pathogenic role as disease severity is reduced in MR1^-/-^ animals or with MR-1 antagonist isobutyl 6-formyl pterin (65). However, oxazolone can induce a very severe colitis with a systemic inflammatory response resembling sepsis, thus likely to reflect only the most severe forms of disease, making it challenging to draw parallels with the full spectrum of IBD (66). A recent study has shown that whilst a significant expansion is seen in the Tc17 population during active CD, this is largely due to induction of conventional T cells and not MAIT cells. Disease associated Tc17 cells acquire a distinct phenotype (CD6^high^, CD39, CD69, PD-1, CD27^low^). MAIT cells were the major IL-17 producing CD8+ population in blood during health or remission but not active CD, when their frequency declines in blood but is maintained in tissue (67). A subpopulation of predominantly CD8+ Crohn’s-associated invariant T (CAIT) cells have also been described, resembling NKT type II cells and enriched in blood of CD patients concurrently with decline in MAIT cell clonotypes. This could represent a compensatory expansion of an innate-like cell population, but the role of these cells is yet to determined (68). IL-17 is reported to have an additional protective role in the intestinal mucosa (69, 70) and clinical trials with anti-IL-17 therapy (secukinumab) have lacked efficacy in CD (71). IL-12 and IL-18 are upregulated in the intestinal mucosa of CD patients and polymorphisms of IL-23R, NLRP3, IL-18R and IL-12B2 significantly associate with CD implicating these cytokines in its pathophysiology (72). It remains to be seen if phenotypic changes reported in tissue MAIT cells in IBD are an epiphenomenon or directly implicated in pathogenesis. Taken together with barrier disruption and dysbiosis in IBD, MAIT cell may need to balance conflicting roles; their capacity to produce IL-17 and engage a tissue repair program following exposure to microbial antigens provides homeostatic capacity (35), however in a dysbiotic landscape with high antigen burden and pro-inflammatory cytokine co-stimulus a pro-inflammatory program may be engaged (36).

### The gut-liver axis and chronic liver diseases

In animal models the liver has been shown to remain sterile in the presence of an intact intestinal mucosa, with immune responses to gut commensals confined to the mesenteric lymphoid system. In the context of infection or inflammation the liver acts as an immunological ‘firewall’, clearing bacteria or their derived products breaching intestinal or vascular barriers (73). MAIT cells are the dominant population of innate like T cells in the liver (up to 50% of CD3^+^ cells). They are adapted for tissue homing (high expression of CXCR6 and CCR6) and poised for host defense (CD69, HLA-DR, CD38^high^). Compared with mucosal barrier MAIT cells, their steady state responses are less skewed to type 17 functionality and require IL-7 licensing for sustained IL-17 production following TCR ligation (74). This distinction may be driven by the lack of interaction with commensal organisms in health. Below we consider changes to the microbiome in prevalent chronic liver diseases in which MAIT cells have been implicated.

Fatty liver diseases associated with alcohol, obesity or metabolic syndromes continue to grow in prevalence. High fat diet and alcohol induced dysbiosis can disrupt host-microbe interactions through metabolic dysregulation and mucosal barrier disruption. In this setting MAIT cell activating bacteria and microbe derived metabolites can translocate to the liver (75). Non-alcoholic fatty liver disease (NAFLD) affects approximately 40% of all adults worldwide and can range from benign hepatic steatosis to progressive non-alcoholic steatohepatitis (NASH) (76). Peripheral blood MAIT cells are depleted in NAFLD, with their enrichment in the liver (77). A negative correlation has been reported between circulating MAIT cell frequency and serum glycated hemoglobin (HbA1c), gamma-glutyl transferase or total triglycerides. These cells upregulate PD-1, CCR5 and CXCR6 (77). Upon stimulation, an IL-4 dominant response is seen over IFN-γ and TNF production. *In vitro*, activated MAIT cells can induce monocyte/macrophage differentiation into a M2 phenotype (77). Furthermore, in a mouse model of NASH, induced with methionine and choline deficient diet, MAIT cells localize to the liver and display a Th2 profile (IL-4^+^ and IL-10^+^ > IFN-γ^+^ cells) in wild-type animals. In the MR1^-/-^ counterparts there was greater steatohepatitis with an accompanying increase in the proportion of CD11c^+^ pro-inflammatory M1 macrophages relative to CD206^+^ M2 macrophages suggesting a potentially protective role for MAIT cells (77). However, in the context of fibrosis, a more pro-inflammatory and fibrogenic function has been proposed given the observation of more progressive carbon tetrachloride (CCl_4_) induced liver fibrosis in animal models using MAIT cell enriched (Vα19TCRTg) over MAIT cell deficient (MR1^-/-^) mice (78). Both in alcoholic and non-alcoholic fatty liver disease MAIT cells have been shown to accumulate in liver fibrotic septa and demonstrate increased proliferative capacity with upregulation of Ki-67 (78). With progression to cirrhosis these cells are closely positioned to fibrogenic myofibroblasts; *in vitro* co-culture experiments have shown MR1 and contact-dependent mitogenic effects of MAIT cells on human myofibroblasts (78). Blood MAIT frequency declines with liver fibrosis, and remaining MAIT cells are activated (CD25 and CD69 high). Interestingly long-term prophylactic antibiotic therapy (norfloxacin or rifaximin) is significantly associated with less decline in MAIT cell frequency and lower CD25 expression (78). The human gut microbiome in NAFLD and NASH is devoid of Bacteroidetes, with expansion of Prevotella and Porphyromonas species. In NASH the proportion of ethanol producing bacteria also increases (75). Rifaxamin is the most widely used antimicrobial prophylaxis agent for the prevention of hepatic encephalopathy in end stage liver disease. It reduces bacterial translocation, has anti-inflammatory properties and modulates the microbiota, specifically reducing the abundance of harmful bacteria (e.g. *Klebsiella, Streptococcus, Clostridium*) relative to probiotic organisms (e.g. Bacteroides) (79).

Intestinal microbiota has also been linked to susceptibility in alcoholic liver disease (ALD) (80). The gut microbiome shifts with disease progression from steatohepatitis, through to fibrosis then cirrhosis and with patterns of alcohol consumption (binge drinking vs chronic consumption). The microbiome had been largely characterized in animal models, with limited human studies (81). Overall, there is lower abundance of Bacteroidetes and a higher proportion of Enterobacteriaceae and Proteobacteria (75, 81). Humanized germ-free mice, following transplantation of intestinal microbiota from patients with severe alcoholic hepatitis, develop hepatitis and intestinal barrier impairment (80). In a murine model of acute binge on the background of chronic alcohol exposure, MAIT cells became depleted in the intestine, liver and lung. Liver and lung MAIT cells upregulated CD69, IFN-γ, TNF and the transcription factor T-bet. It is worth noting that female animals were exclusively used in this study, given the variable effects of ethanol consumption and differences in microbiome driven by sex (82). Alcohol exposure reduced intestinal microbiome beta diversity (a measure of community variation between animals), with resultant reduction in riboflavin production capacity. Adoptive transfer of cecal microbiota to antibiotic pre-treated alcohol naïve mice produced a decline in pulmonary and hepatic MAIT cell frequency, with increased IFN-γ^+^/T-bet^+^ cells at these sites and TNF^+^ cells in the lung. This change in MAIT cell frequency could be abolished with antibiotic therapy following alcohol exposure. Serum levels of intestinal fatty acid binding protein (iFABP), a biomarker of intestinal epithelial damage, and bacterial 16S rRNA gene copies were elevated following alcohol exposure. *In vitro* treatment of human MAIT cells with this serum reduced viability, driving apoptotic death and upregulation of CD38, IFN-γ and granzyme B. Direct ethanol exposure did not produce the same effect (82). Similarly, blood MAIT cells are depleted in patients with severe alcoholic hepatitis and alcohol related cirrhosis with altered transcriptional programming (reduced RORγt and PLZF expression), activated phenotype (CD69^high^) and altered responses (reduced IL-17 and granzyme B with *E. coli* challenge). Plasma endotoxin and D-lactate (measure of gut permeability) are increased with ALD. Faecal extracts derived from patient with ALD reproduce the abnormal MAIT cell phenotype *in vitro* with accelerated cell death, upregulation of CD69 and HLA-DR and diminished antibacterial capacity (reduced IFN-γ, TNF, IL-17, granzyme B and perforin with *E. coli* infection) (83).

The role of MAIT cells in chronic liver disease pathogenesis is yet to be fully defined; indeed, their deficiency in advanced disease could contribute to increased susceptibility towards systemic infection, particularly with shifts seen in the microbiome. Conversely these changes in the context of wider inflammatory and pro-fibrotic signals could account for MAIT cell phenotypes observed in cirrhosis. It is not known if MAIT cells directly interact with and shape the microbiome in homeostasis, by extension further studies are needed to determine if following barrier disruption MAIT cells are bystanders responding to changes in their microenvironment or lose an innate capacity to support commensal communities.

### Obesity and metabolic syndromes

There is evidence that changes in the gut microbiome may contribute to the development of obesity (84), with murine and human studies suggesting gut microbe-derived lipopolysaccharide and translocating gut bacteria may contribute to systemic inflammation in obesity (85–87), which can drive insulin resistance associated with the development of type 2 diabetes (88). Obesity is associated with a decrease in overall circulating MAIT cells (89–91), but an increase in circulating, activated, IL-17 producing MAIT cells, which may be correlated with translocated bacteria such as Bacteroidetes (89, 90). Obesity is also associated with reduction in glycolytic metabolism, mTORC1 signaling, and SLC7A5 aa transport in circulating MAIT cells (92). IL-17-producing and Granzyme B^+^ MAIT cells are increased in omental adipose tissue in human obesity (89, 91). Moreover, whilst circulating MAIT cells are deficient in obesity, weight loss after bariatric surgery is associated with both a restoration of circulating MAIT cell numbers, and an increased diversity of the gut microbiome, including increased in Bacteroidetes and Fusobacteria (93).

Most human studies are correlative, but in murine models, with leptin deficient or high-fat diet-fed mice, MAIT cells were likewise decreased in peripheral blood and ileal and epididymal adipose tissue, due to a shift towards a pro-apoptotic phenotype, with relative increase in activated, IL-17-producing MAIT cells (88). These changes were correlated with a decreased expression of MR1 ligands and of riboflavin pathway genes within the cecal microbiome. These MAIT cells may have been contributing to insulin resistance, as MR1^-/-^ mice had greater insulin sensitivity to oral insulin tolerance testing. Adipose MAIT cells were also contributing to enhanced pro-inflammatory cytokine and chemokine signaling in adipose tissue, and to a reduction in FoxP3+ Treg and a shift towards M1 rather than M2 polarization of macrophages (88). The presence of MAIT cells was also associated with decreased gut epithelial barrier integrity, measured by FITC-dextran translocation and by expression of tight junction proteins, which would favor bacterial translocation. Thus, these data suggest that MAIT cells promote inflammation in obesity. Might these changes in MAIT cells also affect the gut microbiome in a deleterious manner? In the same mouse model fecal transfer was performed into C57BL/6 mice from MAIT-deficient MR1^-/-^ or MAIT over-expressing Vα19^+/-^ mice, and showed MAIT cells promoted a microbiome lower in Bifidobacteriaceae and Lactobacteriaceae, and these changes were again associated with a decrease in gut epithelial barrier integrity and in frequencies of mucosal Treg, innate lymphoid cell(ILC)2 and ILC3 cells, which implies the MAIT-dependent effects on the microbiome are not simple epiphenomena, but can have significant immunological effects.

### Haematopoietic stem cell transplantation

Allogenic HSCT can offer curative therapy for a wide range of hematological disorders but can be complicated by inappropriate immune reconstitution, resulting in inflammatory sequelae such as acute or chronic graft versus host disease (GVHD), and increased risk of infection (94). Post-transplantation intestinal microbiome is associated with survival and complications related to HSCT (95–97). Through their interaction, the intestinal microbiome and mucosal T cells, particularly human MAIT cells, are thought to play a protective role post-transplant (30, 94, 98). In a murine major MHC (class I and II) mismatched allogenic stem cell transplant model, MAIT cell frequency was higher in tissue compared with peripheral blood (41). MAIT cells preferentially localized to the colon, producing high concentration of IL-17A to maintain barrier integrity and limit alloantigen presentation after bone marrow transplantation. By comparison IL-17A^-/-^ and MR1^-/-^ animals displayed relatively accelerated GVHD, associated with altered fecal microbiota and downregulation of tight junction proteins claudin 4 and claudin 8 (41). In human HSCT, a diverse intestinal microbiome early after transplant is associated with a higher MAIT cell frequency (30), reduced incidence of acute GVHD (99, 100) and improved survival (100). Circulating Vδ2 cell frequency correlated with MAIT cells and intestinal alpha diversity, a measure of species diversity and abundance (100). Specifically, a higher abundance of Bacteroidetes was seen with higher MAIT cell numbers, whereas abundance of Firmicutes was associated with fewer MAIT cell (100). *Blautia* spp. abundance is also predictive of MAIT reconstitution (98). No increased abundance of the riboflavin biosynthesis pathways is however observed between individuals based on MAIT cell frequency (100). Single cell RNA sequencing of peripheral blood MAIT cells revealed a pro-inflammatory phenotype, upregulating genes linked to effector function (GNLY, PRF1, CCL4) and migration (ITGB2) with concurrent downregulation of NfκB signaling inhibitors (NFKBIA, TNFAIP3). Vδ2 cells also display a complementary activated phenotype. As expected, a tissue repair signature was not detected in peripheral blood MAIT cells (100). High MAIT cell frequency in infused grafts is linked to higher abundance of intestinal flora post-transplant and lower incidence of acute GVHD. Following the onset of GVHD, circulating MAIT cell frequency declines as these cells become activated (upregulating CD69, CXCR3, CXCR4 and transcription factors RORγt and T-bet). This coincides with a decline in riboflavin synthesising gut microbiota, perhaps reflecting a component of microbial regulation by MAIT cells in health (99). These studies provide insight into the interdependence of MAIT cells and the microbiome in a unique setting of immune reconstitution in human adults. However, due to the lack of tissue MAIT cell profiling several questions remain unanswered including whether MAIT cells can repopulate tissues at similar baseline frequencies and if their phenotype is altered upon their return thereby affecting homeostatic functions, barrier integrity and microbiome composition.

### Chronic infections and immunodeficiency

In a similar pattern to the systemic inflammatory conditions discussed above, chronic infection with HIV (101–104) and HCV (105–107) cause a decline in circulating MAIT cell frequency (108) which cannot be fully restored with treatment (101, 109) and is associated with intestinal dysbiosis (108). Circulating MAIT cells upregulate perforin and granzyme B in HIV and HCV infection, with high CD69 and PD-1 expression in viral co-infection. Following *ex vivo* stimulation with *E. coli* there is impaired IFN-γ, TNF and IL-17 generation in presence of mono- or co-infection, compared with health. Virally-infected patients have relatively low endotoxin core antibodies, implying a diminished capacity to control translocating bacteria. There is a compositional change in the fecal microbiome with higher abundance of Bacteroidetes and lower abundance of Firmicutes following viral infection. *Bacteroides* spp. abundance has been shown to correlate positively with MAIT cell frequency but negatively with TNF production following bacterial challenge, whereas Firmicutes abundance negatively correlated with PD-1^+^ MAIT cell frequency (108). Gut dysbiosis is not reversed with antiretroviral therapy (ART) in HIV (110) and there is partial recovery following HCV eradication (111) except in cirrhotic patients (112). The lung microbiome is also less diverse in HIV and only partially restored with ART (113). Intriguingly, compared with blood, lung MAIT cells better retain function and transcriptional features in HIV, including a tissue repair capacity (48). These studies further support a role for the microbiome in shaping MAIT cell function; circulating MAIT cells are likely reflective of the state of the gut microbiome given its association with barrier disruption and potential microbial translocation into circulation, whereas at different barrier sites (e.g. lung) the local microbiome has the capacity to uniquely modify tissue resident MAIT cell phenotype. It also raises further questions regarding the requirements for reconstitution of MAIT cells following their depletion – how essential are individual constituents of the microbiome and their capacity for riboflavin synthesis? What other co-stimulatory signals are required and potentially deficient in these viral infections? And, is complete re-population of tissue with MAIT cells restricted by a temporal window as in mice? These questions need to be addressed in human studies to best translate therapeutic strategies for re-establishing tissue homeostasis with MAIT cells.

### The respiratory microbiome and MAIT cells

The respiratory tract encompasses the upper (anterior nares, nasal passages, paranasal sinuses, nasopharynx, oropharynx, and laryngeal segment proximal to the vocal cords) and lower (larynx distal to vocal cords, trachea, small airways, and alveoli) tracts. The total surface area of the airways is approximately 70m^2^ making it the second largest barrier site after the gut mucosa in human (114). Culture independent methods of microbial detection have dispelled the long-held theory of lung sterility and instead demonstrated the presence of diverse communities of microbiota in the lower airway (115). MAIT cells are enriched in the airways and form the largest population of antibacterial T cells in the lungs (116); they express tissue residency markers (CD69 and CD103) and display polycytotoxic potential (117). Thus, MAIT cells occupy a prime position to support pulmonary immunity, and there has been growing interest in studying their role in airways diseases. Airways inflammation and common treatments (corticosteroids and antimicrobials) significantly modify the local microbiome and alter barrier function, therefore careful consideration of the interdependence between MAIT cells and the microbiome is crucial when studying pulmonary pathology as discussed below.

### The upper airway

The oropharynx harbors a diverse microbiome characterized by the genera *Streptococcus, Neisseria, Rothia*, and anaerobes, including *Veillonella., Prevotella* and *Leptotrichia* (114). It is thought to be the primary source of lung microbiota, introduced through subclinical microaspiration (118). MAIT cells are present in the buccal mucosa (up to 50% CD8αα T cells), displaying a tissue resident effector memory phenotype (CD69, CD103, HLA-DR and PD-1 high) and IL-17 skewed response to PMA-ionomycin stimulation (46). One study has considered oromucosal MAIT cell function in relation to the microbiome in apical periodontitis, characterized by inflammation of the periodontal tissue leading to translocation and dissemination of opportunistic organisms. In this condition a MAIT cell signature appears in affected tissue with increased MAIT TCR, TNF, IFN-γ and IL-17A transcripts compared to healthy adjacent gingiva. There is an expansion of riboflavin producing taxa in the local microbiome and using a sparse partial least squares discriminant analysis the authors report several bacterial genera negatively correlated with MAIT TCR and IL-17A transcripts (119). Whilst this is an interesting approach, a large variation in abundance and diversity of bacterial taxa was observed in this study within a small population (n=25), thus there is limited power to derive conclusions regarding mechanisms of microbiome-MAIT cell interaction.

The nasopharynx is similarly enriched with MAIT cells (47) but is home to a unique microbiome compared with the oropharynx and lower respiratory tract. As a transition site between keratinized squamous epithelium and stratified squamous epithelium it hosts skin colonizers from the genera *Staphylococcus, Propionibacterium* and *Corynebacterium* alongside *Moraxella, Corynebacterium, Dolosigranulum, Haemophilus* and *Streptococcus* (114). As seen in the oropharynx, sinonasal MAIT cells possess a tissue resident effector memory phenotype. They have been linked to disease severity in allergic rhinitis with nasal polyposis; in this condition MAIT cell appear more activated with CD38 upregulation and IL-17A skewed response to stimulation. Both markers correlate with disease severity (47). Allergic rhinitis and nasal polyposis are often accompanied by asthma. In these conditions, changes to the nasopharyngeal microbiome have been reported with expansion of MAIT cell activating organisms belonging to Bacteroidetes and Proteobacteria taxa in asthma (120) and predominantly Firmicutes, Proteobacteria and Actinobacteria in chronic rhinosinusitis with nasal polyposis. Interestingly in children, nasal *Corynebacterium* sp. and *S. epidermidis* abundance is associated with absence of pet allergen sensitization (121); *Corynebacterium* have been shown to negatively correlate with inflammatory gene expression in the nose (122) whilst *S. epidermidis* can engage a tissue repair program in skin resident murine MAIT cells (21). It is tempting to speculate that the microbiome in health supports a homeostatic phenotype in MAIT cells whereas disease driven changes to the microbiome supply the antigenic and co-stimulatory signals to sustain an inflammatory IL-17 dominant response. It is yet to be determined if MAIT cells can in fact interact with the microbiome in this manner and indeed if any potentially pathogenic activity can be reversed by manipulating the microbial community.

### The lower airway

The healthy adult lung microbiome is dominated by the genera *Prevotella, Veillonella*, and *Streptococcus* (123). These belong to the phyla Bacteroidetes and Firmicutes, both stimulators of MAIT cells, with a stronger capacity in the former group (31). In lung diseases there are notable changes to the microbiome likely provoked by host inflammatory responses often leading to increased airway wall permeability and mucus production modifying growth conditions (124–126). A sustained bidirectional relationship between the mucosal immune system and disordered respiratory microbiota is likely to be a key driver of disease progression. We propose that the shift in community membership towards species with greater MAIT cell antigenic load, immunogenicity and capacity for epithelial disruption can overwhelm MAIT cell homeostatic barrier defenses, particularly with numeric and functional deficiencies seen in these conditions as exemplified below.

Airways diseases carry a huge global burden; asthma is the commonest chronic respiratory disease affecting 262 million people worldwide (127) and chronic obstructive pulmonary (COPD) is the third leading cause of mortality (128). These conditions have uniquely disordered airway microbiome but share a pulmonary MAIT cell deficiency proportionate to inhaled corticosteroid (ICS) dose (116, 129), a mainstay of therapy with increasing disease severity.

Asthma is a clinically and immunologically heterogenous condition. Treatment approaches in asthma have been revolutionized by identifying and targeting ‘treatable traits’ (130), notably in type-2 cytokine (IL-5, IL-4, IL-13) mediated eosinophilic airways inflammation for which novel biologics have emerged (131, 132). ‘T2-low’ non-eosinophilic disease is refractory to corticosteroids and existing biologics (130). It affects ~30% of severe asthmatics (133), and is associated with airways neutrophilia alongside high IL-17 (133) expression, and may be driven by chronic bacterial airways infection (118, 134–138). Epithelial barrier disruption is central to pathogenesis, regardless of inflammatory phenotype (139). An altered naso-/hypopharyngeal microbiome can predict development of allergic asthma in childhood (134, 140). Circulating MAIT cell frequency at 1 year of age is associated with reduced risk of asthma diagnosis within the first 7 years of life and a Th1 dominant response in CD4^+^ T cells (141). In pediatric asthma, small studies have reported increased frequency of IL-17^+^ MAIT cells in bronchoalveolar lavage (142) and blood (143) of patients presenting with severe exacerbations, however no comparisons were made with health when sampling the lower airway (142). In adult disease *Haemophilus influenzae* has emerged as the commonest potentially pathogenic organism in the airway, associated with sputum neutrophilia and altered microbial diversity, namely reduction in *Streptococcus, Gemella* and *Porphyromonas* taxa (135, 136, 144, 145). In a large bronchoscopy study no evidence of increased IL-17A in serum, sputum or BAL was found in asthma nor was there an increase in IL-17^+^ T cell populations (Th17 or γδT cells). This study identified a striking deficiency in MAIT cell frequency with increasing disease severity and ICS use (116). Corticosteroid exposure has been demonstrated to impair MAIT cell IFN-γ responses *in vitro* to non-typeable *H. influenzae* (NTHi), the most prevalent strains linked to exacerbations of airways diseases (129). Sputum MAIT cells are CD69 and PD-1 high, and peripheral blood cells from patients with asthma are skewed towards IL-17/TNF production (over IFN-γ) following activation. IL-7 levels in sputum and serum are elevated in neutrophil dominant airways inflammation and induces greater IL-17 response to PMA-Ionomycin *ex vivo* stimulation (146). MAIT cell frequencies have been reported to correlate with NK, ILC1, ILC2, ILC3 cells in severe asthma, declining with airflow obstruction (reduction in FEV1%) (147). In a murine model of allergic airway inflammation using Alternaria inhalation, MAIT cells are proposed to repress ILC2 driven inflammation and airway hyperresponsiveness *via* expression of interleukin-4-induced gene 1 (IL4I1) (148). In asthma, MAIT cells are thus depleted and exposed to a Proteobacteria dominated microbiome in the setting of epithelial barrier disruption. Translocation of bacterial antigen and products may account for the activated MAIT cell phenotype however ICS-disabled anti-bacterial responses could cause susceptibility to airways infection seen in severe asthma. Moreover, their absence could deprive the airway of tissue repair and type-2 immune regulatory mechanisms. The lines of causality in severe treatment refractory asthma, particularly with dominance of pathogenic organisms in the airway and neutrophilic infiltration, are very complex. It likely involves barrier dysfunction and mucosal immune disarmament; in the case of MAIT cells it is yet to be determined if a pathogenic role can be ascribed or if these cells are stripped of their homeostatic antibacterial function due to the wider spanning inflammatory landscape or indeed treatment. Future studies therefore need to examine their function in well characterized patient cohorts and compare tissue resident cells in disease affected and unaffected locations with due consideration to the local microbiome.

COPD is also heterogenous in its pathogenesis and manifests with predominantly neutrophilic airways inflammation, mucus hypersecretion, emphysema and variable vascular dysfunction (149). Unlike asthma, alterations in airway microbiome appear late with more severe disease. The microbiome declines in diversity, it is depleted of Bacteroidetes with a relative expansion in Proteobacteria, particularly *Haemophilus* and *Moraxella* (123, 150, 151). MAIT cells are depleted in blood and endobronchial biopsies of corticosteroid treated (but not ICS naïve) COPD patients (129). A further study has reported MAIT cell depletion in blood with enrichment in lung parenchyma and accumulation around alveolar epithelial cells in less clinically severe disease, however a limitation of these data is the lack of reporting on ICS use in lung tissue donors (152). There is a higher frequency of IL-17^+^ lung MAIT cells in COPD compared to health, and blood MAIT cells generate IL-17 (over IFN-γ) following PMA-ionomycin stimulation *in vitro* with diminishing magnitude of response seen with worsening airflow obstruction (152). Lower MAIT cell frequencies are associated with elevated serum C-reactive protein (CRP) levels (153) and increased frequency of exacerbations requiring hospitalization (154). At the time of exacerbation peripheral blood MAIT cells are activated with upregulation of CD38 and LAG-3 (154). The above studies hint towards an impaired antibacterial defense with MAIT cell deficiency in COPD sufficient to cause clinical exacerbation events as disease severity increases. The relationship with ICS in COPD and asthma is important and raises the question whether we should supplement such therapies with counteractive strategies to boost barrier MAIT cell frequencies either directly with ligand (5-OP-RU) or indirectly by manipulating the microbiome (e.g. with probiotics). Further work is also needed to understand the significance of MAIT cell derived IL-17 in the setting of neutrophilic airways inflammation – is it a sufficient signal to perpetuate neutrophil recruitment or are MAIT cells attempting to re-establish tissue homeostasis? These questions need to be considered in the context of the microbiome and dysregulated epithelium (155).

### The skin microbiome and MAIT cells

The skin microbiome is critical for homeostasis and shaping of the mucosal immune system. As previously discussed, cutaneous commensals have been shown to induce a homeostatic tissue repair program and functionality in murine skin MAIT cells. In human skin, MAIT cells are a tissue resident population upregulating skin homing marker CLA and CD103 and not enriched in common skin lesions (except dermatitis herpetiformis) (156). The colonizing microbial population in human skin is composed of a core group of species and variation is seen with change in topology, introduced by structures such as hair follicles, sebaceous glands and ducts, alongside environmental factors (20, 157). The core phyla comprising the epidermal microbiome are Actinobacteria (up to 50% of organisms), Firmicutes, followed by Proteobacteria and Bacteroidetes. The gut microbiome has also been implicated in shaping skin health given common cutaneous manifestations of GI disorders such as IBD and coeliac disease. The mechanisms underlying gut-skin microbial interactions are not known but it is postulated that gut dysbiosis and resultant systemic inflammation or intestinal microbial translocation may contribute to disrupted skin homeostasis (157).

Skin dysbiosis is recognized in multiple chronic inflammatory conditions including psoriasis, atopic dermatitis, rosacea and acne vulgaris. In psoriasis there are complex patterns of change in microbial composition with variation in reporting between studies (158). Within psoriatic lesions increased *S. aureus* abundance and decreased *S. epidermidis* abundance have been observed (158), this pattern is also reported in atopic dermatitis (157). MAIT cells can mount a cytotoxic response to *S. aureus* infected dendritic cells in an IL-12 reliant and partially MR-1 dependent manner, with IFN-γ and Granzyme B upregulation (159). Thus, they are equipped to provide cutaneous antibacterial defenses against *S. aureus*. MAIT cells originally garnered interest in psoriasis as a source of IL-17A, which is a key driver of inflammation; but Teunissen et al. have shown that the majority of IL-17A^+^CD8+ T cells are in fact conventional CD8+ T cells rather than MAIT cells (160). In atopic dermatitis abundance of *S. aureus* induces a host transcriptomic signature characterized by upregulation of genes encoding antimicrobial factors, tryptophan metabolites, immune activation (IL1B, CCL2, CCL19) and Th2 signaling mediators (IL4R, IL5, IL13, PI3, TNFRSF4, CCR4) (161). Interestingly in a murine model of atopic dermatitis MAIT cells have been implicated in eosinophil activation and recruitment of IL-4/-13 producing type 2 effector cells in a MR1 dependent fashion (162). The skin therefore reflects another major barrier site at which the local microbiome may shape not only antimicrobial type 1 immunity exercised by MAIT cells but also influence the regulation of type 2 allergic inflammation. There is a relative paucity of human data examining the balance between tissue repair and proinflammatory functionality in MAIT cells in the context of primary skin disorders or cutaneous manifestations of systemic inflammatory disorders. Murine and human *in vitro* data have shown successful harnessing of MAIT cells to accelerate wound healing through TCR ligation (21, 36). It is yet to be seen if this capacity can be utilized as an add-on therapy to address barrier disruption in skin diseases through direct application of MAIT cell ligand or manipulation of the skin/gut microbiome with targeted topical or oral probiotics respectively. This approach may even be relevant within the female genital tract, as an extension of the skin barrier and a mucosal site at which MAIT cells are enriched (49).

## Discussion and future directions

A common theme within this expanding body of data is that dysbiosis at any mucosal surface can affect MAIT cell frequency and function, both locally and at distant sites, with dysbiosis typically associated with a decrease in circulating MAIT cell frequencies, but a relative increase in activated, IL-17-producing, pro-inflammatory MAIT cells. Conversely changes in MAIT cell frequency have also been shown to drive changes in the host microbiome, with evidence these can in turn have direct immunological consequences. Given the incredible diversity of bacteria within human microbiomes, full understanding of these complex interactions with the mucosal immune system is hard to achieve, and further research focusing *in vivo* on changes in defined microbial species and their consequences for the host immune response are warranted.

Nonetheless, already there is scope for investigating the potential for therapeutic manipulation of the microbiome or MAIT cell compartment.

Manipulation of the gut microbiome could be achieved using simple MR1 ligands. Indeed an *in vivo* murine study showed conceptual proof of principle using exogenous oral administration of the synthetic inhibitory ligand acetyl-6-FP in obese mice. This reduced obesity-associated ileal inflammation and decreased MAIT cell IL-17 production (88). Furthermore, this ligand also led to alteration of the gut microbiome, inducing an increase in Bacteroidetes abundance. From these data a picture emerges of MAIT cells whose functions are very context dependent, and likewise the intent of therapeutic manipulation will be context dependent. In the situation of obesity, changes in the microbiome lead to reduced tissue MAIT cell frequencies, likely through increased apoptosis, but also to increased activation of MAIT cells promoting type 1 biased chronic inflammation, and consequent metabolic dysfunction. In such a situation therapeutic inhibition of MAIT cells has the potential to reduce dysbiosis, improve gut integrity and ameliorate systemic inflammation and metabolic dysfunction.

Conversely in contexts of chronic mucosal infection or epithelial damage, therapeutic stimulation of MAIT cells would be anticipated to promote beneficial antibacterial responses and activate tissue repair programs promoting epithelial wound repair. The use of activating ligands would require selection of synthetic molecules with much greater stability than naturally-occurring 5-OR-RU which degenerates rapidly in aqueous solution.

A key aspect of MAIT cell biology which remains to be elucidated is how it is determined whether the effects of MAIT cell stimulation lead to a dominant pro-inflammatory antimicrobial response, or to a more homeostatic tissue repair activity favoring restoration of epithelial integrity. It is likely that a critical determinant of MAIT cell response is the integrity of the epithelial barrier itself. A damaged epithelium will release a number of factors which may influence MAIT cells directly, including alarmins, such as IL-33, whose receptor IL1R1/ST2 is highly expressed on lung MAIT cells during acute bacterial infection (35). MAIT cells also highly express IL17RE in mice and humans (35), the receptor for IL-17C. IL-17C is abundantly released by epithelia after stimulation by IL-1β, TNF, various pathogens or through cell damage *via* TLR2 and TLR5, promoting a proinflammatory, Th17 response from T cells (163). A damaged epithelium will also allow translocation of pathogens into proximity to the MAIT cells, directly furnishing danger signals such as lipopolysaccharide, which will activate membrane TLR2, which is particularly highly expressed on stimulated human MAIT cells (35). Conversely, in situations where riboflavin producing commensals are abundant, but the epithelium is intact, the MAIT cell will receive TCR stimulation alone, promoting a dominant homeostatic tissue repair response (35, 36). Therefore, approaches to manipulate MAIT cell biology may need to simultaneously target these danger signal pathways in addition to the MAIT TCR, for instance combining inhibition of TLR2 or IL17RE with inhibitory MAIT cell ligands to reduce inflammation. Theoretically MAIT TCR ligands could be used sequentially or at different stages of an inflammatory response, favoring antagonistic ligands with or without additional immunosuppressants to reduce overt inflammation, followed subsequently by agonistic ligands to support restored barrier integrity. Extensive modelling in experimental systems would be required before clinical trials could be considered.

An alternative approach to therapeutic manipulation of MAIT cells would be altering the microbiome could be altered directly by the administration of probiotics which favor MAIT ligand producing microbiomes, and might thereby enhance the overall riboflavin-synthetic capacity of the fecal microbial community. The net effects of such an intervention are, however, unpredictable, due to complex symbiotic relationships between microbes, such that increasing riboflavin availability might, paradoxically, favor the growth of otherwise less dominant species which lack this synthetic pathway. The potential impact of a modulated MAIT microbiome to impact the host immune response was demonstrated in work by Toubal et al. (88) which showed that feces from MR1-/- mice triggered more MAIT cell activation than feces from MR1 sufficient mice, suggesting MAIT cells are able to differentially sense microbiome assemblages which differ in riboflavin synthesis. The use of probiotics is controversial as a large proportion of bacteria fail to survive the gastric and upper GI environment, or to become significantly established amongst the complex, diverse microbiome of the lower GI tract which compete for the same niche. Nonetheless it could be possible to engineer bacterial species to over-express riboflavin pathways to enhance MAIT cell activation of the entire microbial assemblage, which might for instance help accelerate reconstitution of mucosal immune cell populations after HSCT or HIV. The expected clinical effect of these will need to be determined using *in vivo* models because the ultimate effect on MAIT cell activation will be influenced by a number of factors which are hard to predict from *in vitro* experiments, including differing efficiencies of Rib pathway enzymes, interactions between riboflavin producing and riboflavin scavenging species and the extent to which other microbial factors trigger concomitant innate immune signaling.

Within the airways administration of MAIT cell ligands might be manipulated to enhance immune responses during vaccination, as a component of aerosolized vaccines, or as an adjunctive treatment for persistent microbial infection. Indeed, preliminary work has explored this in the context of chronic infection with *Mycobacterium tuberculosis (M.tb)* (164). During acute infection administration of 5-OP-RU did not enhance protective responses, but surprisingly rather delayed T cell priming through mechanisms dependent on MAIT cells and TGF-β. However, conversely, during chronic infection intrapulmonary 5-OP-RU administration drove a 30-fold MAIT cell expansion and an IL-17A-dependent 10-fold reduction in pulmonary bacterial loads. Of note this protective effect was local to the mucosa and did not affect bacterial load in the liver, implying the potential to manipulate mucosal MAIT cell populations selectively.

In human airways disease, long term antibiotic therapy with macrolide antibiotics has been shown to reduce inflammation and exacerbations of asthma (165). The mechanism is unknown, but it is postulated that reduction in bacteria such as *Haemophilus influenzae* colonizing the respiratory mucosa may lead to amelioration of mucosal inflammation. It is known that MAIT cells are deficient in airways diseases (116, 129) so studies using direct airway tissue sampling should explore whether the MAIT cell populations are restored by antibiotics and whether this is associated with restoration of a homeostatic rather than proinflammatory MAIT cell phenotype.

Simpler than both these approaches, and more amenable to very simple clinical trials would be assessment of the utility of topical application of MAIT cell ligands or riboflavin-synthesizing commensal bacteria of low invasive capacity to accelerate wound healing, as has already been demonstrated in mice with H2-M3 restricted commensal bacteria (37) and with topical 5-OP-RU (21).

In the decade since the first MR1 ligands were discovered (1) our knowledge of MAIT cell biology has expanded rapidly. Though there remain many unanswered questions, we should expect to see the field start to progress to the next stage of clinical translation over the next 5 years.

## Author contributions

MJ and TH jointly conceived the review, conducted the literature review, and drafted the manuscript. TH created the figures. All authors contributed to the article and approved the submitted version.
